# Fatty Acid Synthase as a Potential Metabolic Vulnerability in Ocular Adnexal Sebaceous Carcinoma

**DOI:** 10.3390/cancers18020349

**Published:** 2026-01-22

**Authors:** Autumn Berlied, Isabella Boyack, Andre Vieira, Maria Gonzalez-Perez, Vikas Kumar, Cornelia Peterson

**Affiliations:** 1 Department of Comparative Pathobiology, Tufts University Cummings School of Veterinary Medicine, North Grafton, MA 01536, USA; autumn.berlied@tufts.edu (A.B.); isabella.boyack@tufts.edu (I.B.); 2 Department of Biochemistry & Molecular Biotechnology, University of Massachusetts Chan Medical School, Worcester, MA 01605, USA; andre.vieira@umassmed.edu (A.V.); mariapaz.gonzalez-perez@umassmed.edu (M.G.-P.); vikas.kumar5@umassmed.edu (V.K.); 3 Mass Spectrometry Facility, University of Massachusetts Chan Medical School, Worcester, MA 01605, USA; 4 Tufts Center for Vision Research, Tufts Medical Center, Boston, MA 02111, USA

**Keywords:** Meibomian gland (MG), MYC, C75, proliferation, differentiation, apoptosis, mass spectrometry, fatty acid, saturation, ceramide

## Abstract

**Background**: MYC dysregulation is frequent in ocular adnexal sebaceous carcinoma (SebCA), an aggressive malignancy without precision therapy. Fatty acid synthase (FASN) expression and lipid metabolism are commonly perturbed in high-MYC-expressing tumors; however, the role of MYC and FASN in the coregulation of lipid biosynthesis and tumorigenesis in SebCA is unknown. **Methods**: The aim of this study was to characterize the effects of FASN inhibition on MYC expression, oncogenic processes, and lipid profiles in vitro, using non-neoplastic human Meibomian gland epithelial cells (HMGECs) and three primary SebCA cell lines, and in vivo, utilizing a conditionally *MYC*-overexpressing mouse model. **Results**: FASN inhibition reduced cell viability, proliferation, and clonogenicity and altered the saturation profile of fatty acids across multiple lipid classes. The relative saturation of ceramides was the most variable between treatment conditions. *MYC* overexpression in the murine Meibomian gland promoted proliferation while suppressing sebaceous differentiation. Subsequent topical FASN inhibition further reduced sebaceous differentiation, attenuated *PLIN2* expression, and induced apoptotic cell death. **Conclusions**: Collectively, these findings suggest that MYC expression in SebCA is responsive to FASN inhibition. Pharmacologic targeting of FASN reveals a metabolic vulnerability that may serve as a target for future therapeutic development.

## Simple Summary

This study aimed to better understand ocular adnexal sebaceous carcinoma, a rare and aggressive cancer of the eyelid that currently has no targeted treatments. Using cultured tumor cells and a genetically modified mouse model, we determined that this cancer may be vulnerable to alterations in the fat synthesizing enzyme fatty acid synthase. Fatty acid synthase and the gene *MYC* work together to change how the tumor cells make and use fats, which are important for tumor progression. By blocking fatty acid synthase, cell growth was slowed, and cell death was triggered, uncovering a potential weakness in the cancer’s metabolism. These findings open the door to developing new therapies that could improve outcomes for patients facing this potentially fatal condition.

## 1. Introduction

Ocular adnexal sebaceous carcinoma (SebCA) is an uncommon but phenotypically aggressive neoplasm, most frequently arising from the Meibomian gland (MG), for which the mechanistic drivers remain poorly understood. Conventional chemotherapies, including mitomycin-C and 5-fluorourocil, have demonstrated efficacy against primary SebCA cell lines, resulting in improved differentiation and diminished proliferative potential [1]. In the absence of precision therapies, wide surgical resection remains the standard of care for SebCA, often yielding both anatomical and functional morbidity [2,3].

Genomic and molecular profiling have demonstrated copy number gains at the *c-MYC* locus and upregulated MYC protein expression in a subset of SebCAs [4]. Activation of the *MYC* transcriptional program induces sterol regulatory element-binding protein-1 (SREBP-1) expression, and, collectively, these two transcription factors regulate lipid metabolism enzymes and promote tumorigenesis [5,6]. SREBP-1 preferentially activates fatty acid (FA) and triglyceride assembling genes, chiefly, fatty acid synthase (*FASN*), to facilitate de novo FA synthesis [5,7]. These free FAs serve as substrates for the generation of the more complex lipids which comprise phospholipid membranes [5].

Exploiting FASN expression in various tumor models has widely demonstrated a metabolic vulnerability and suggested potential promise for therapeutic targeting in high-MYC-expressing tissues [5,8]. The impact of FASN inhibition on MYC overexpression or alterations in the lipidome in the context of SebCA initiation and progression is unknown. The objective of this study was to establish the role of FASN activity on the cellular biology and metabolic features of both non-neoplastic human MG epithelial cells (HMGECs) and three primary ocular adnexal SebCA cell lines. Furthermore, we sought to characterize the morphometric, histochemical, and molecular features in the *MYC*-overexpressing murine MG in response to topical FASN inhibition.

## 2. Materials and Methods

### 2.1. In Vitro Pharmacologic FASN Inhibition

Early passage (P4-22) HMGECs (ATCC, Manassas, VI, USA) were maintained in Keratinocyte Serum-Free Medium (KSFM; Gibco, Waltham, MA, USA) supplemented with bovine pituitary extract (BPE; 50 µg/mL), human recombinant epidermal growth factor (EGF; 5 ng/mL), and normocin (100 µg/mL; InvivoGen, San Diego, CA, USA) as previously described [9]. Three primary human ocular adnexal sebaceous carcinoma cell lines (SebCA01, SebCA02, SebCA03; P4-24) were maintained in CRC-conditioned medium containing human EGF (10 ng/mL; Gibco), cholera toxin (8.4 ng/mL; Sigma-Aldrich, Burlington, MA, USA), hydrocortisone (25 ng/mL; Sigma-Aldrich), and the ROCK inhibitor Y-27632 (5 µM; Selleck Chemicals, Houston, TX, USA) as previously described [1]. All cells were incubated at 37 °C with 5% CO_2_. To assess the effects of FASN modulation in vitro, all cell lines were treated with the FASN inhibitor C75 (Tocris Biosciences, Minneapolis, MN, USA), which is a competitive inhibitor of malonyl-CoA at the ß-ketoacyl synthase (KS) binding domain of FASN [10]. Catalytic inhibition of the KS domain blocks a critical component of FA elongation [11]. DMSO was used as a vehicle control.

### 2.2. In Vitro Proliferative and Clonogenic Assessments

All cell lines were seeded in 96-well plates at a density of 500 cells/well to assess proliferative responses. Cells were incubated with 10× serial dilutions of an FASN inhibitor or vehicle control (*n* = 3 wells/concentration/time point) for three days. A CyQuant MTT assay (ThermoFisher, Waltham, MA, USA) was performed according to the manufacturer’s instructions, and the sample absorbance (optical density: OD) was read at 570 nm. Percent viability was calculated using the following formula:

% viability = (OD_modulator-treated_ − OD_media only_)/(OD_vehicle control_ − OD_media only_) × 100.

Proliferation was evaluated following 24 h incubation with the FASN inhibitor (C75 10 µM) in all cell lines using the Click-iT Plus EdU Imaging Kit (ThermoFisher) with the addition of EdU (10 μM) for the last four hours of drug incubation per the manufacturer’s instructions. Quantification of both EdU- and Hoechst-positive cells was performed by counting the number of positive nuclei for each stain in four 20× fields, and the proliferation rate for each 20× field was calculated using the following equation:

% proliferation = (number of EdU-positive cells/number of Hoechst-positive cells) × 100.

To assess the clonogenic responses of both non-neoplastic and neoplastic meibocytes to FASN inhibition, all cell lines were seeded into 6 well plates at a density of 500 cells/well. Cells were incubated with 100 nM C75 or DMSO for 14 days (*n* = 3 wells/treatment/cell line). Drug-treated media were then aspirated, and the plates were washed with 1× PBS. Plates were then fixed with 10% neutral buffered formalin for 20 min before being stained with 0.01% (*w/v*) crystal violet for 30 min at room temperature (RT). Plates were washed with deionized water and allowed to dry before being imaged using an LI-COR Odyssey imaging system (LICORbio; Lincoln, NE, USA). Stained colonies were counted using Image J software (v. 1.54k; National Institutes of Health, Bethesda, MD, USA) as previously described [12].

A SimpleStep ELISA Kit (catalog # ab323521; Abcam, Waltham, MA, USA) was used per the manufacturer’s instructions to evaluate c-MYC expression following 24 h incubation with an FASN inhibitor (C75 10 µM; *n* = 3 wells/treatment/cell line). Cell lysates were diluted to 100 µg/mL, the sample absorbance (OD) was read at 450 nm, and the c-MYC concentration was determined using the standard curve.

### 2.3. Immunocytochemistry and Apoptosis Assessments

Cells were seeded in 8-well chamber slides at a density of 2.2 × 10^6^ cells/well. Following a 24 h incubation with C75 10 µM, FASN expression was evaluated using a primary antibody against FASN (catalog # PA5-22111; Invitrogen, Carlsbad, CA, USA) diluted to 1:400 with 3% BSA + 2% normal goat serum (NGS) in 1× PBS + 0.1% Tween (PBS-T) and incubated overnight at 4 ° C. Slides were then incubated with goat anti-rabbit, Alexa Fluor 555 (catalog # A-27039; Invitrogen), at a dilution of 1:1000 with 3% BSA + 2% NGS in PBS-T for 60 min at RT. An H-Score (0–300) was then calculated by multiplying the staining intensity of the target protein (0: absent, 1: weak, 2: moderate, 3: strong) by the percentage (by decile) of positively labeled-cells (*n* = 3 wells/treatment/cell line). ReadyProbes Reagent F-actin probes (catalog # R37112 and R37110; ThermoFisher) were diluted in wash buffer and incubated for 30 min at RT prior to DAPI staining and mounted as previously described [13]. Apoptosis in treated cell lines and in formalin-fixed paraffin-embedded (FFPE) sections of murine eyelid were evaluated using the Scientific Click-iT TUNEL Assay for In Situ Apoptosis Detection with the Alexa Fluor Kit (ThermoFisher) per the manufacturer’s instructions. Quantification of both TUNEL and DAPI-positive cells was performed by counting the number of positive nuclei for each stain in four 40× fields, and the apoptotic rate for each 40× field was calculated using the following equation:

% apoptosis = (number of TUNEL-positive cells/number of DAPI-positive cells) × 100.

### 2.4. Lipidomic Analysis

Lipid profiles were evaluated following 24 h incubation with C75 10 µM in all cell lines. Cell lysis was performed using 1× M9 buffer composed of potassium phosphate monobasic (20 mM), sodium phosphate-dibasic (48 mM), sodium chloride (8.5 mM) in deionized water. The lipids were extracted using the Folch Method with a mixture of chloroform–methanol (2:1). After drying, the samples were reconstituted in 200 µL of acetonitrile:2-propanol–water (65:30:5 *v*/*v*/*v*) and pipetted into glass HPLC vials. Reconstituted samples (30 µL) were then injected into a Thermo Q Exactive HFX and coupled with a Thermo Vanquish UHPLC system. The analysis was performed with a Thermo C18 Hypersil Gold column (50 mm × 2.1 mm, 1.9 µm) with a 31 min gradient. The flow rate was set to 250 µL/min, using a solvent composition of solvent A: water–acetonitrile (60:40 *v*/*v*), 10 mM ammonium formate (NH_4_COOH), 0.1% formic acid, and solvent B: isopropyl alcohol–acetonitrile (90:10 *v*/*v*), 10 mM ammonium formate (NH_4_COOH), 0.1% formic acid. The gradient method began with 32% B over 0–1.5 min; 32–45% B from 1.5 to 4 min; 45–52% B from 4 to 5 min; 52–58% B from 5 to 8 min; 58–66% B from 8 to 11 min; 66–70% B from 11 to 14 min; 70–75% B from 14 to 18 min; 75–97% B from 18 to 23 min; 97% B up to 25 min; 97–32% B from 25 to 25.5 min; 32% B is maintained until 31 min for column equilibration [14].

The following parameters were used for the HPLC and MS conditions: column oven temperature was maintained at 50 °C and autosampler was set 10 °C with mobile phase flow rate at 250 μL/min and MS scan range between *m*/*z* 200 and 2000. The capillary temperature was set at 325 °C, the sheath gas flow rate was set at 45 units, the auxiliary gas flow was set at 10 units, the source voltage was 3.2 kV, and the AGC target was 10^6^. The acquisition was conducted using full-scan data-dependent MS2 (ddMS2) mode. For MS1 profiling, scans were run at a resolution of 120 k. MS2 analyses were performed using five scan events, where the top five ions were chosen from an initial MS1 scan. For fragmentation, a normalized collision energy of 35 was used [14].

Compound Discoverer (v. 3.4.0.1183, ThermoFisher) was used to search the data against five libraries containing different metabolites and lipids. The specialized Lipid Maps database (https://www.lipidmaps.org/) was used as primary search tool to apply the data filters and select the best identified lipid candidates, and six other libraries (Thermo mzVault, LipidBlast, mzCloud, ChemSpider-NIST Spectra, PubChem, and Nature Chemistry) were used to further evaluate the veracity of the identified lipid species. Raw data were exported as csv files, compounds were sorted by name (e.g., PC 21:0_13:1) and chemical formula (e.g., C_42_H_82_O_8_P) into one of the following seven lipid classes: triacylglycerols (TG), phosphatidylcholines (PC), phosphatidylethanolamines (PE), lysophosphatidylcholines and lysophosphatidylethanolamines (LPC/LPE), cholesterol esters (CEs), sphingomyelins (SM), and ceramides (Cer), and relative quantification was determined for each cell line and treatment condition. The AUC for each sample (*n* = 8) was used to normalize the individual lipid compound abundance as a percentage of the total AUC of each cell line. Lipid compounds were subsequently arranged by class and saturation and sorted by treatment before the relative abundance of saturated and unsaturated compounds was determined using R Studio (v. 4.5.1, Posit, Boston, MA, USA; R script included in Supplementary Materials).

### 2.5. RNA Isolation, Reverse Transcription, and Quantitative PCR (qPCR)

RNA was extracted from homogenized murine eyelids (*n* = 3 or 4/group) using a combination TRIzol (ThermoFisher) protocol and an RNeasy Kit (catalog #74104; Qiagen, Germantown, MD, USA), and in vitro samples (*n* = 3/treatment/cell line) were subject only to the RNAeasy Kit following a 24 h incubation with C75 10 µM or DMSO. RNA was stored at −80 °C until further processing. Reverse transcription was performed using a SuperScript VILO Kit (ThermoFisher). Quantitative polymerase chain reaction (qPCR) was conducted using the TaqMan Gene Expression Assays (ThermoFisher) and a QuantStudio 3 System (ThermoFisher). Expression of *MYC*, *FASN*, and *PLN2* was quantified using 2^−ΔΔcT^ normalized to *polR2α* using predesigned probes: Hs00153408_m1 (human *MYC*), Mm00487804_m1 (murine *MYC*), Hs01005622_m1 (human *FASN*), Mm00662319_m1 (murine *FASN*), Hs00605340_m1 (human *PLIN2*), Mm00475794_m1 (murine *PLIN2*), Hs00172187_m1 (human *polR2α*), and Mm01309448_m1 (murine *polR2α*) (ThermoFisher).

### 2.6. Modulation of MYC and FASN Expression In Vivo

The Tufts University Institutional Animal Care and Use Committee (IACUC) approved all animal protocols utilized in this study (G2023-01; renewal: G2025-102). Animal experiments were performed in accordance with the guidelines for the Use of Animals in Ophthalmic and Vision Research of the Association for Research in Vision and Ophthalmology (ARVO), and the information regarding in vivo experiments reported in this manuscript is in adherence with the ARRIVE 2.0 Guidelines [15]. Both female and male transgenic (TG) mice on a C57B6 background and expressing the human *MYC-2* cDNA fused to the hormone-binding domain (ERTM) of a mutant murine estrogen receptor in a keratin 14 (K14) expression cassette (K14MycER) and their wildtype (wt) littermates were used for in vivo evaluations [16]. At the time of weaning (P21), mice were screened for the presence of the transgene by PCR of distal tail tissue with the following primers: F: 5′-TACTCTGAGTCCAAACCGGGC-3′; R: 5′-AGCCTGGTAGGAGGCCAGCTTCTCTGA-3′, as previously described [16].

Adult mice (*n* ≤ 5/cage) were maintained in Nexgen 500 individually ventilated cages (Allentown Inc., Allentown, NJ, USA) in a temperature and humidity-controlled (70 ± 2.0 °F; 30–70%), 12 h day/night light cycle environment with food (irradiated Teklad Global 18% Rodent Diet; Inotiv, Indianapolis, IN, USA) and water ad libitum. Both breeding females and adolescent mice were also provided DietGel GEM dietary supplement (Clear H_2_O, Westbrook, ME, USA) in the peri-gestational and peri-weaning periods, respectively.

MYC induction was achieved in TG mice (P42-70) through once daily intraperitoneal administration of 4-hydroxytamoxifen (4-OHT; Sigma–Aldrich; 10 mg/kg/day) dissolved in ethanol and corn oil for three days. Both wt (*n* = 13) and TG (*n* = 14) mice were treated once daily with unilateral topical application of C75 (20 mg/kg/day) to the eyelid skin for three days beginning 24 h after the last dose of 4-OHT. Vehicle-treated (DMSO) contralateral eyes served as controls. Mice were humanely euthanized 48 h following their last administration of C75 or vehicle. A subset of mice (*n* = 2/genotype) was administered a single dose of 5-Bromo-2′-deoxyuridine (BrdU; Sigma-Aldrich) intraperitoneally (100 mg/kg in sterile 0.9% saline) two hours prior to euthanasia. Eyelids with periocular skin and globes were collected and either fixed in 4% PFA for 48 h and then stored in 1× PBS prior to processing for paraffin embedding or freezing for embedding in OCT or were immediately homogenized in RIPA buffer (ThermoFisher) or TRIzol for subsequent protein and RNA extraction.

### 2.7. Histology, Morphometry, and Immunohistochemistry

Hematoxylin and eosin (H&E)-stained whole mount sections of FFPE (*n* = 3 or 4/group) and Oil-Red-O-stained sections (StatLab, McKinney, TX, USA) of frozen, OCT-embedded murine eyelid tissue (*n* = 2/group) were utilized to visualize the morphology and lipid content of MGs, respectively. Whole-slide images (WSIs) of H&E-stained sections were obtained using a VS200 Research Slide Scanner (Olympus, Westborough, MA, USA) at 40×, and the cross-sectional area of meibocytes was determined using the arbitrary line tool in the OlyVIA (Olympus, v.4.2, build 31689) as previously described [13]. Meibocytes without clear sebaceous differentiation, circumferentially distinct cytoplasmic borders, and visible nuclei in the plane of section were excluded from morphometric measurements.

Routine fluorescent immunohistochemistry was performed on OCT-embedded murine eyelid using standard techniques. BrdU expression was evaluated using an anti-BrdU antibody (catalog # ab6326; Abcam) at a dilution of 1:200 with 3% BSA + 2% NGS in 1× PBS-T and incubated overnight at 4 °C. Slides were then incubated with goat anti-rat, Alexa Fluor 488 (catalog # A-11006; Invitrogen) at a dilution of 1:1000 with 3% BSA + 2% NGS in PBS-T for 60 min at RT. DAPI staining and mounting was performed as previously described [13]. Quantification of both BrdU and DAPI-positive cells was performed by counting the number of positive nuclei for each stain in four 40× fields, and the proliferation rate for each 40× field was calculated using the following equation:

% proliferation = (number of BrdU-positive cells/number of DAPI-positive cells) × 100.

Routine chromogenic immunohistochemistry was performed on FFPE murine eyelid using standard protocols. Antigen retrieval was performed using sodium citrate buffer at 95 °C for 10 min. Differentiation was evaluated using an anti-adipophilin (ADFP, catalog # PA5-79830: Invitrogen) antibody at a dilution of 1:150 with 2.5% normal equine serum overnight at 4 °C, followed by a universal biotinylated anti-mouse/rabbit IgG (catalog # PK-7200; Vector Laboratories, Newark, CA, USA) for 60 min at RT. Antigen was visualized using a 3,3′-Diaminobenzidine (DAB) chromogen (ThermoFisher), hematoxylin counterstain, and standard brightfield microscopy. MYC expression was evaluated following antigen retrieval in TE buffer (pH 9.0) at 95 °C for 20 min using the HRP-DAB IHC Detection Kit (Abcam) and an anti-c-MYC (Y69, Abcam) antibody at a dilution of 1:50 for 60 min at 37 °C.

### 2.8. Statistical Analyses

Percent viability, number of colonies, proliferation and apoptosis rates, FASN H-scores, relative transcript expression, and cross-sectional area of murine meibocytes were evaluated using two-way ANOVAs with Dunnett’s and Sidak’s post hoc tests for multiple comparisons relative to controls. Statistical analyses were performed using Prism GraphPad (v. 10.4.2; San Diego, CA, USA) (α = 0.05).

## 3. Results

### 3.1. In Vitro Viability, Clonogenicity, and Proliferation

**Figure 1:**
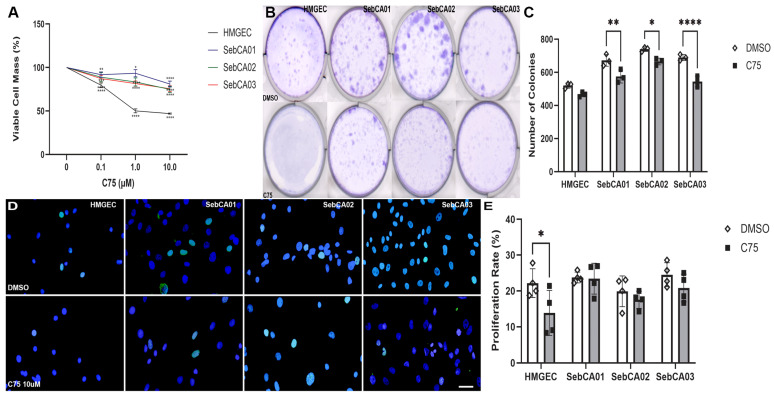
FASN-mediated viability, clonogenicity, and proliferation in vitro. (**A**) HMGECs (black) were the most sensitive to C75 treatment across all concentrations evaluated, while SebCA01 (blue) was the most resistant to changes in viability resulting from incubation with C75. * *p* < 0.05, ** *p* < 0.01,*** *p* < 0.001, **** *p* ≤ 0.0001. (**B**) The number of anchorage-dependent colonies formed was reduced following a 14 d incubation with C75. (**C**) Quantification of stained colonies demonstrated the most significant reduction in clonogenicity in SebCA03 in response to C75 treatment. * *p* < 0.05, ** *p* < 0.01, **** *p* ≤ 0.0001. (**D**) Fewer EdU-positive nuclei (yellow green, merged) in FASN-inhibited cells were observed relative to DMSO control. EdU: Alexa Fluor 488, green. Hoechst: blue. Scale bar: 25 µm for all panels. (**E**) Quantification of EdU-positive nuclei demonstrated a significant reduction in mean proliferation rate in C75-treated cells relative to DMSO only in HMGECs. * *p* < 0.05.

Cellular viability, clonogenic potential, and proliferative responses were evaluated in HMGECs and three primary SebCA cell lines in response to in vitro FASN inhibition with C75. All cell lines exhibited reductions in the percentage of viable cells in response to a three-day incubation with all concentrations of C75 (Figure 1A). In response to 14-day incubation with C75, anchorage-dependent colony formation was significantly reduced in all three SebCA lines (SebCA01: *p* = 0.0011; SebCA02: *p* = 0.0101; SebCA03: *p* ≤ 0.0001), but this reduction did not reach significance in HMGECs (*p* = 0.0777; Figure 1B,C). Proliferation, determined by EdU-immunolabeling, was significantly decreased in C75-treated HMGECs (*p* = 0.0272), but was not different between FASN-inhibited and vehicle-treated SebCA lines (SebCA01: *p* > 0.999; SebCA02: *p* = 0.8764; SebCA03: *p* = 0.5908; Figure 1D,E).

### 3.2. FASN-Mediated Protein and Transcript Modulation

**Figure 2:**
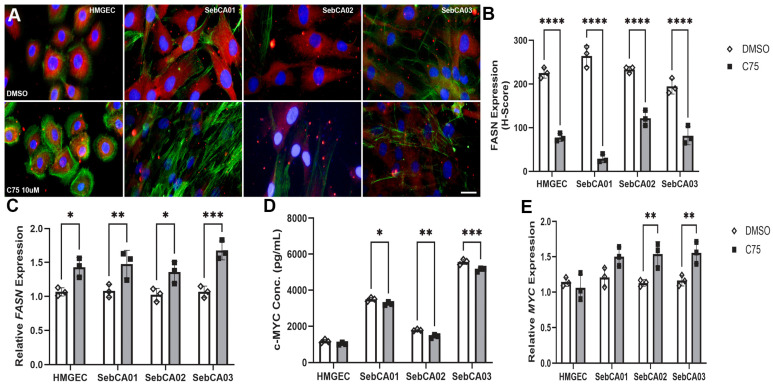
Differential protein and transcript expression following FASN inhibition in vitro. (**A**) Cytoplasmic FASN expression (Alexa Fluor 555, red) was reduced in C75-treated cells relative to vehicle control (DMSO) across all cell lines. Phalloidin: Alexa Fluor 488, green. DAPI: blue. Scale bar: 10 µm for all panels. (**B**) Semi-quantitative evaluation of FASN expression by H-score determination revealed significant downregulation in response to C75 treatment in all cell lines. **** *p* ≤ 0.0001. (**C**) Relative *FASN* expression was significantly upregulated in all cell lines following C75 treatment. * *p* < 0.05, ** *p* < 0.01, *** *p* < 0.001. (**D**) Assessment of c-MYC protein concentration by ELISA showed significant decreases in MYC concentration in all SebCA lines treated with C75 relative to vehicle control. * *p* < 0.05, ** *p* < 0.01, *** *p* < 0.001. (**E**) Relative expression of *MYC* was significantly induced in response to C75 treatment in SebCA02 and SebCA03. ** *p* < 0.01. 2^−ΔΔcT^ was utilized to normalize target transcript expression to *polR2α.*

The efficacy of FASN inhibition on both FASN and MYC expression was evaluated in all cell lines in response to 24 h incubation with C75. FASN expression was attenuated following incubation with C75 (Figure 2A). When immunolabeling was assessed by H scoring, significant downregulation was demonstrated in all cell lines (*p* ≤ 0.0001; Figure 2B).

Relative *FASN* expression was significantly upregulated in all cell lines following FASN inhibition (HMGEC: *p* = 0.0139 SebCA01: *p* = 0.0077; SebCA02: *p* = 0.0250; SebCA03: *p* = 0.0001; Figure 2C). MYC concentration was significantly attenuated in all three SebCA lines following C75 treatment when measured by ELISA (HMGEC: *p* = 0.2938; SebCA01: *p* = 0.0272; SebCA02: *p* = 0.0013; SebCA03: *p* = 0.0002; Figure 2D). Relative *MYC* expression was significantly upregulated in C75-treated SebCA02 and SebCA03 relative to vehicle control (HMGEC: *p* = 0.9197 SebCA01: *p* = 0.0582; SebCA02: *p* = 0.0065; SebCA03: *p* = 0.0085; Figure 2E).

TUNEL labeling was increased in FASN-inhibited cells relative to vehicle controls (Supplementary Figure S1A). Apoptotic rates were significantly (*p* ≤ 0.0001) increased in all C75-treated cells relative to DMSO control (Supplementary Figure S1B).

### 3.3. Lipid Profiling in FASN-Inhibited Meibocytes

Lipid profiles and the saturation status of seven lipid classes were characterized using mass spectrometry of FASN-inhibited HMGECs and SebCA cell lines. We aimed to determine whether dysregulated expression of FASN resulted in metabolic reprogramming in non-neoplastic, low-MYC-expressing HMGECs and high-MYC-expressing SebCA cells.

**Figure 3:**
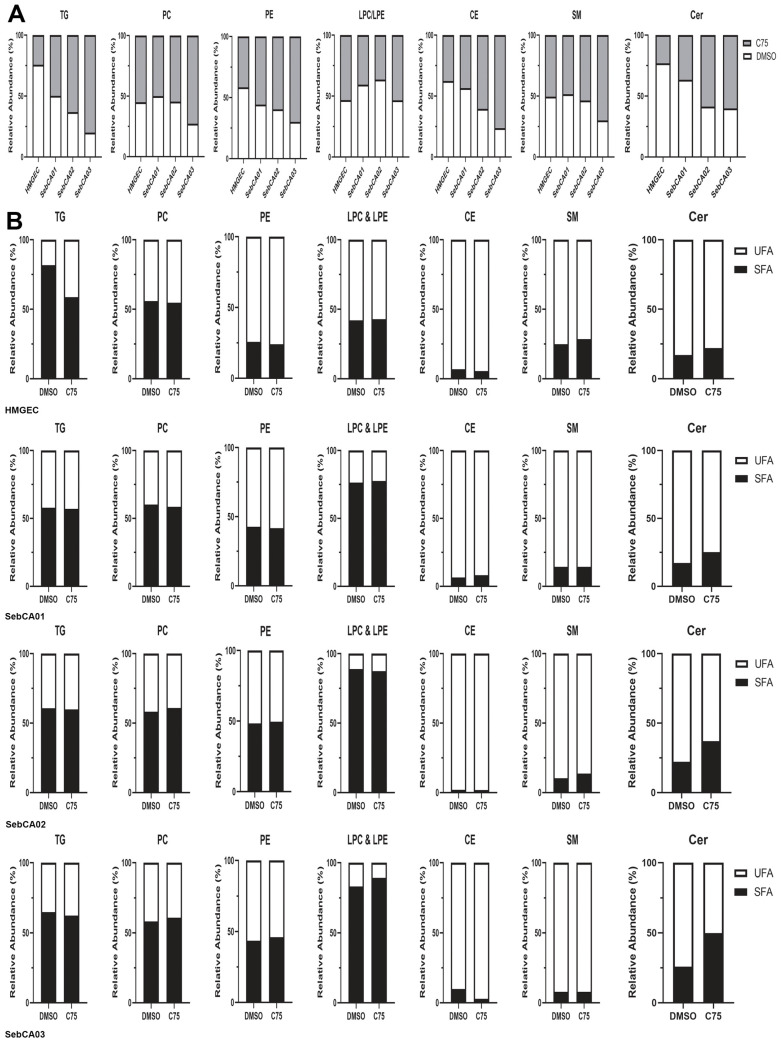
Lipidomic alterations secondary to FASN inhibition in vitro. (**A**) The relative abundance of each lipid class across cell lines in response to incubation with an FASN inhibitor. (**B**) The relative abundance of saturated (SFAs) and unsaturated fatty acids (UFAs) from each lipid class across treatments in each cell line. TG: triacylglycerol; PC: phosphatidylcholine; PE: phosphatidylethanolamine; LPC and LPE: lysophosphatidylcholine and lysophosphatidylethanolamine; CE: cholesterol ester; SM: sphingomyelin; Cer: ceramide.

The relative abundance of triacylglycerols (TGs) was increased in all cell lines in response to incubation with C75 in SebCA02 and SebCA03 cells (Figure 3A). Collectively, TGs isolated from all cell lines and under all treatment conditions exhibited greater than 50% saturated fatty acids (SFAs) (Figure 3B).

Evaluated glycerophospholipid classes included phosphatidylcholines (PCs), phosphatidylethanolamines (PEs), lysophosphatidylcholines (LPCs), and lysophosphatidylethanolamines (LPEs). The abundance of both PCs and PEs tended to be relatively unchanged across cell lines in response to C75 treatment. PCs demonstrated a relatively balanced ratio between SFAs and unsaturated fatty acids (UFAs) in all cell types regardless of treatment, demonstrating approximately 50% saturation. LPC and LPE saturation profiles were balanced across treatments but tended to be higher in SebCA cells than in HMGECs. CEs, representing FA compounds from the sterol lipid category, were also isolated from all cell lines. The relative abundance of CEs increased most in SebCA03 cells following incubation with C75 relative to DMSO, and the SFA:UFA ratios were low in all cell lines under all treatment conditions.

Evaluated sphingolipid classes included SMs and ceramides. The SM SFA:UFA ratio was relatively low in all cell lines; however, the ratios demonstrated variation between treatments. The SFA:UFA ratio of ceramides increased in all cell lines in response to FASN inhibition relative to DMSO. The individual lipid compounds isolated from these seven lipid classes with a relative abundance of at least 0.1% are presented for HMGECs and SebCA cells in Supplementary Figures S2–S5, respectively.

### 3.4. In Vivo Responses to FASN Inhibition

**Figure 4:**
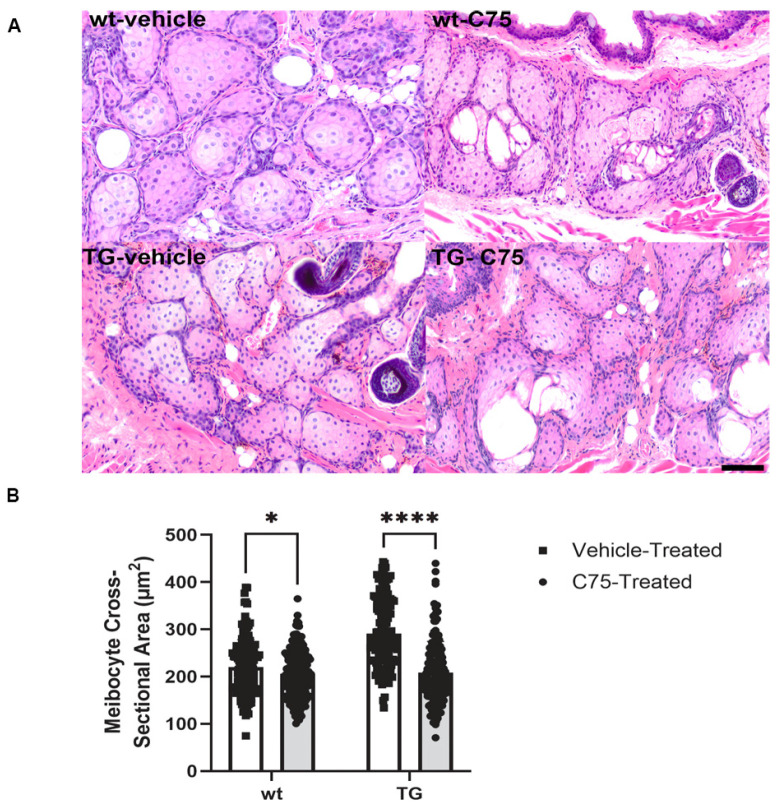
Histopathological and morphometric alterations resulting from MYC and FASN modulation in vivo. (**A**) Representative H&E-stained FFPE sections of murine tissues. Conditionally *MYC*-overexpressing transgenic (TG) Meibomian glands exhibited basal meibocyte hyperplasia which was reduced following topical FASN inhibition. The cytoplasmic volume was reduced in both TG and wildtype (wt) mice in response to topical administration of C75. Scale bar: 50 µm. (**B**) The mean cross-sectional area C75-treated meibocytes was significantly reduced relative to vehicle control. * *p* < 0.05, **** *p* ≤ 0.0001.

Transgenic (TG) conditionally *MYC*-overexpressing mice and their wildtype (wt) littermates were utilized to examine the effects of FASN inhibition on the MG in vivo. Following a course of intraperitoneal 4-OHT, adult wt and TG mice were administered topical C75 or vehicle control. No apparent gross changes to the ocular surface or adnexa were observed in any mice. FASN-inhibited wt glands demonstrated more frequent pyknotic nuclei relative to contralateral vehicle treatment, suggesting increased rates of apoptosis. *MYC*-overexpressing TG mice, subsequently treated with vehicle, demonstrated proliferation of the outer basaloid meibocytes (basal hyperplasia) and a mild reduction in the cytoplasmic volume of the more central, well-differentiated meibocytes (Figure 4A). FASN-inhibited TG mice demonstrated attenuation of the hyperplastic response noted in vehicle-treated animals. Quantification demonstrated a significant reduction in meibocyte cross-sectional area in both wt (206.0 ± 51.6 µm^2^; *p* = 0.0429) and TG (208.4 ± 65.9 µm^2^; *p* ≤ 0.0001) eyelids treated with C75 relative to vehicle-treated wt (220.1 ± 58.1 µm^2^) and TG (291.1 ± 71.5 µm^2^) eyelids (Figure 4B). However, the differences in cross-sectional area between TG and wt mice did not reach significance (*p* = 0.0521).

**Figure 5:**
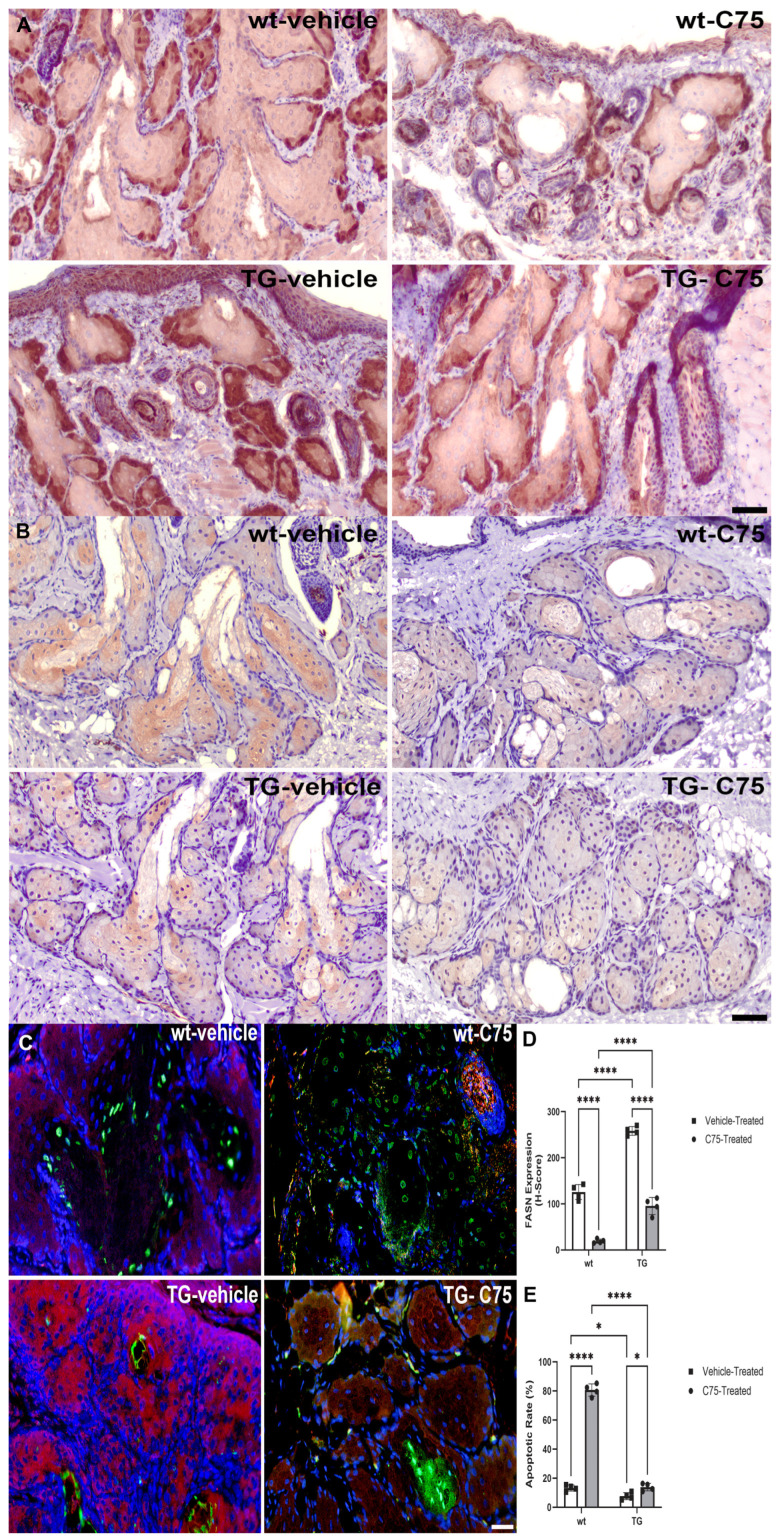
MYC modulatory, differentiative and apoptotic effects of FASN inhibition in vivo. (**A**) Representative images of chromogenic and fluorescently immunolabeled FFPE sections of murine tissues. Vehicle-treated *MYC*-overexpressing transgenic (TG) Meibomian glands exhibited pronounced upregulation of MYC (DAB: brown) relative to wildtype (wt) littermates and compared to contralateral C75-treated eyelids. Scale bar: 50 µm. (**B**) Adipophilin (DAB: brown) expression was diminished in TG mice relative to wt littermates and in C75-treated eyelids relative to vehicle controls. Scale bar: 50 µm. (**C**) *MYC*-overexpressing TG glands exhibited upregulated FASN expression (Alexa Fluor 555, red) relative to wt littermates and when compared to C75-treated eyelids. C75-treated eyelids exhibited increased distribution of apoptotic cells (TUNEL: Alexa Fluor 488, green) when compared to vehicle controls. DAPI: blue. Scale bar: 50 µm. (**D**) FASN immunolabeling was assessed by H scoring, demonstrating significant downregulation in response to C75 treatment in both wt and TG mice and in wt when compared to TG littermates. **** *p* ≤ 0.0001. (**E**) Quantification of TUNEL-positive meibocytes demonstrated a significant increase in mean apoptotic rates in C75-treated eyelids relative to contralateral controls and in wt mice relative to TG. * *p* < 0.05; **** *p* ≤ 0.0001.

Proliferation and differentiation were assessed in both FFPE and frozen sections of murine eyelid. Oil-red O (ORO) staining and BrdU immunolabeling were performed on frozen, OCT-embedded sections. ORO staining of cytoplasmic lipid droplets was reduced in *MYC*-overexpressing TG mice relative to wt littermates and in FASN-inhibited glands relative to vehicle control (Supplementary Figure S6A). BrdU immunopositivity was most intense in the expanded basaloid population of meibocytes from vehicle-treated TG eyelids, with less intense and less widely distributed immunolabeling observed following C75 treatment and in wt animals relative to TG (Supplementary Figure S6B). Proliferation rates were significantly higher (*p* ≤ 0.0001) in TG animals (vehicle: 70.3 ± 4.7%, C75: 35.2 ± 4.6%) relative to wt littermates (vehicle: 35.9 ± 4.5%, C75: 24.9 ± 4.7%), and C75 treatment significantly (wt: *p* = 0.0056, TG: *p* ≤ 0.0001) impaired proliferation relative to contralateral controls (Supplementary Figure S6C). MYC expression was suppressed in both C75-treated TG and wt eyelids when compared to contralateral vehicle controls (Figure 5A). Adipophilin expression was evaluated in FFPE tissue to confirm the ORO staining demonstrated in frozen sections. Adipophilin expression was suppressed in *MYC*-overexpressing TG animals relative to wt littermates, and, consistent with ORO staining, C75 treatment further diminished sebaceous differentiation relative to vehicle controls (Figure 5B). Finally, assessments of apoptosis and FASN expression were performed, demonstrating intense staining of FASN in *MYC*-overexpressing mice and diminished FASN expression, concurrent with apoptosis induction, in C75-treated eyelids (Figure 5C). FASN expression was significantly suppressed in C75-treated eyelids (*p* ≤ 0.0001; Figure 5D). Apoptotic rates were significantly increased in C75-treated eyelids (wt: 80.7 ± 4.2%; TG: 13.9 ± 2.5%; *p* ≤ 0.0001) relative to contralateral vehicle controls (wt: 13.2 ± 2.1%; TG: 7.6 ± 2.4%; *p* = 0.0189 Figure 5E).

**Figure 6:**
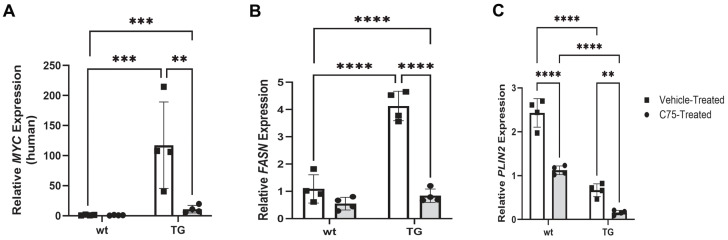
MYC and FASN-mediated differential transcript expression in vivo. (**A**) Relative *MYC* and (**B**) *FASN* expression were significantly upregulated in vehicle-treated TG mice relative to other groups. (**C**) Relative *PLIN2* expression was significantly downregulated in TG mice relative to wt controls and in C75-treated eyelids relative to contralateral vehicle treatment. ** *p* < 0.01, *** *p* < 0.001, **** *p* ≤ 0.0001. 2^−ΔΔcT^ was utilized to normalize target transcript expression to *polR2α.*

Relative expression of the human *MYC* transcript was significantly downregulated in C75-treated TG eyelids (10.9 ± 6.2; *p* = 0.0013) relative to vehicle-treated tissue (117.2 ± 71.9) and when compared to vehicle-treated wt (1.2 ± 0.6) eyelids (*p* = 0.0007) (Figure 6A). Relative *FASN* expression exhibited similar, yet less extreme dysregulation as *MYC*, with significant downregulation in C75-treated TG eyelids (0.8 ± 0.2) relative to contralateral vehicle controls (4.1 ± 0.5; *p* ≤ 0.0001) and when compared to vehicle-treated wt eyelids (0.5 ± 0.2; *p* ≤ 0.0001) (Figure 6B). Expression of perilipin-2 (*PLIN2*), the gene encoding the adipophilin protein, was also evaluated. Relative *PLIN2* expression was significantly downregulated in TG eyelids relative to wt littermates (*p* ≤ 0.0001) and was suppressed in C75-treated eyelids (wt: 1.1 ± 0.1; *p* ≤ 0.0001; TG: 0.2 ± 0.1; *p* = 0.0023) relative to contralateral vehicle controls (wt: 2.4 ± 0.3; TG: 0.7± 0.1) (Figure 6C).

## 4. Discussion

Dysregulated MYC expression is common in many malignancies, and gain-of-function mutations and copy number gains at the *MYC* locus have been documented in ocular adnexal SebCA [4,17]. MYC’s roles in regulating diverse cellular pathways, including the Integrated Stress Response and lipid metabolism, may contribute to sebaceous oncogenesis [13]. De novo lipogenesis is critical in the maintenance of the phospholipid membranes required during tumorigenesis, and both the reciprocal regulation of MYC and FASN on each other and their synergistic effects on lipogenesis are major factors in these metabolic processes of neoplastic cells [5,18]. *MYC* directly promotes *FASN* expression and drives FA metabolism, and pharmacologic FASN inhibition, in turn, feeds back to suppresses MYC protein and transcriptional signatures [19]. This MYC-FASN axis has increasingly been investigated as a strategic target for chemotherapeutic development, with disruption of this pathway effectively rewiring cancer metabolism and inducing ER stress [20,21,22].

Here, we aimed to further characterize the FASN expression and to define a possible mechanism of sebaceous oncogenesis by utilizing a non-neoplastic HMGEC line and three primary SebCA cell lines. A small molecule FASN inhibitor (C75) was employed to evaluate the capacity of FASN to modulate MYC and the resulting cellular phenotypes. We performed MTT and clonogenicity assays in addition to EdU immunolabeling to observe the effects of FASN inhibition on the ability of these cells to survive, form colonies, and proliferate, respectively. All cell lines exhibited reduced viability and clonogenicity when exposed to C75, and HMGECs demonstrated significant reduction in proliferative capacity following incubation in C75. FASN inhibition led to a reduction in perilipin-2 (adipophilin) expression, which is a surrogate marker for sebaceous differentiation [23].

While increased FASN expression fuels membrane biosynthesis through de novo lipogenesis, enhances, or often hyperactivates, oncogenic signaling pathways, and stimulates proliferation, the effects on differentiation are less clearly defined [8,24,25,26]. However, in various epithelial neoplasms including colorectal, ovarian, breast, prostatic, and hepatocellular, upregulated FASN expression has been correlated with poor differentiation, aggressive tumor phenotypes, and worse prognoses [27,28,29,30,31,32]. FASN inhibition reduces proliferation and promotes differentiation, rendering FASN a target of interest for anti-cancer and differentiation-based strategies.

Due to the in vitro phenotypic effects observed in response to FASN inhibition, we then assessed the effects on both protein and transcript expression of MYC and FASN. To evaluate these expression changes, cell lines were again exposed to small molecule inhibitors, and immunolabeling, ELISA, and qPCR were pursued. Reduced MYC protein concentrations were observed in each of the SebCA cell lines in response to FASN inhibition. Inhibition of MYC by C75 suggests either enhanced phosphorylation of Ser62 or Thr58, promoting ubiquitin tagging and proteasomal degradation, or interference with the heterodimerization of MYC with MAX [22,33,34].

FASN inhibition promoted upregulation of *MYC* expression in SebCA02 and SebCA03 and *FASN* expression in all cell lines. These results may, collectively, indicate a compensatory feedback mechanism in response to the C75-induced intracellular lipid deprivation and MYC suppression, promoting nuclear localization of SREBP-1 to restore lipid homeostasis [35]. FASN inhibition is also a potent inducer of metabolic stress, altered translational programming, and dysregulated signaling, any of which may have indirectly enhanced transcription of stress-responsive oncogenes like *MYC* in our tumor cell lines [22]. Specifically, disrupted FASN activity induces malonyl-CoA and activated AMPK, indirectly boosting *MYC* transcription by relieving mTORC1 suppression or engaging unfolded protein response factors, such as ATF4, which drive *MYC* expression as part of survival adaptation programs [36]. In further support of this premise, apoptotic death was enhanced in FASN-inhibited HMGECs and SebCA cells, while the cell lines exhibiting greater basal (DMSO-treated) MYC expression (i.e., SebCA01 and SebcA03) demonstrated a greater resistance to apoptosis, as indicated by immunofluorescent TUNEL expression. C75 has demonstrated pro-apoptotic effects in cultured human tumor cells, contributing to its anti-neoplastic benefits, independent of metabolic cell stress induction [20,37].

The cooperative effects of *MYC*, *SREBF-1*, *FASN*, and peroxisome proliferator-activated receptor gamma (*PPARγ*) in lipid acquisition and storage are well described in cancer biology [38,39,40,41]. However, descriptions of the interplay of these factors in the MG are limited to in vitro studies utilizing HMGECs and analytical assessments of animal and human meibum [42,43,44,45,46]. Collectively, *FASN*, *PPARγ*, and *SREBF-1* orchestrate the process of meibocyte differentiation and meibum synthesis [47]. SREBP-1, in addition to upregulating *FASN*, generates endogenous *PPARγ*-activating ligands [48,49]. These ligands, in turn, further enhance expression of genes driving lipogenic pathways, including *FASN*. The lipid characteristics of meibum are diverse, with several features unique when compared to sebum or other lipid-laden secretions including the ratio of lipid classes isolated, FA chains of extreme length (C32-36), extensive FA and alcohol hydroxylation, complex branching of waxes, and the presence of lipids containing multiple ester bonds [44].

Lipidomic analyses of primary SebCA tissue or cell lines have not been reported, and, here, we aimed to provide a characterization of the lipid profile of our four cell lines in response to FASN inhibition. Triacylglycerol (TG) saturation was stable across treatments in the three SebCA cell lines but was more variable in HMGEC following incubation with C75. The high MYC expression in the three SebCA cell lines may promote multiple metabolic pathways which preserves access to saturated and monounsaturated fatty acids (MUFAs) and permits stability of TG saturation despite our partial FASN inhibition [50]. Conversely low-MYC-expressing non-neoplastic cells (HMGECs) lack this buffering capacity and, consequently, exhibited a decline in saturation due to the selective exclusion of SFAs and MUFAs (i.e., C16:0 palmitic acid and C18:0, stearic acid) from the TG pool [19]. Saturation of glycerophospholipids (PCs, PEs, and LPCs/LPEs), lipid components integral to cell signaling, was relatively balanced across treatment conditions [51].

Cholesterol esters (CEs), lipids critical to the regulation of cell membrane fluidity and a primary component of human meibum, exhibited the lowest saturation ratios of all lipid classes analyzed across all four cell lines [52]. Prior analyses of human MG secretions describe the vast majority (60–80%) of meibum-derived CEs comprising of SFAs [52,53]. HMGECs have been previously criticized as a low fidelity model for the examination of human meibum composition; however, the extraordinarily low saturation profile of CEs isolated from the SebCA cell lines may be attributed to the broad remodeling response to high MYC expression, favoring polyunsaturated fatty acid (PUFA) incorporation into CEs [44,54,55].

Of the sphingolipids isolated from the four cell lines, ceramides exhibited the most variability in relative saturation subsequent to the various treatment conditions, with increasing relative saturation demonstrated following FASN inhibition. Interestingly, the relative saturation of ceramides often reflects a dynamic balance between pro-apoptotic and pro-survival pathways, mediated by the ratio of ceramide to spingosine-1-phosphate (S1P) [56,57,58,59]. High ceramide–S1P ratios indicate the accumulation of saturated ceramides, which are poor substrates for the ceramidase enzyme responsible for the conversion of ceramide to S1P, and demonstrate tumor-suppressive functions including cell cycle arrest, apoptosis, and autophagy [60,61]. Low ceramide–S1P ratios, alternatively, indicate the evolution of tumor cells toward enhanced S1P generation, tilting the ratio toward PUFA utilization and the promotion of migration, angiogenesis, immune evasion, and apoptosis resistance. FASN inhibition redirects available SFAs to ceramide synthesis rather than for other complex lipids, leading to the accumulation of saturated ceramide species and the triggering of apoptosis [62,63,64,65].

Finally, we also evaluated these questions in a conditionally *MYC*-overexpressing mouse model. Meibocyte cross-sectional area was significantly lower in FASN-inhibited eyelids when compared to contralateral vehicle-treated eyelids in both wt and TG mice following systemic 4-OHT induction, without a significant difference between genotypes. Interestingly, the magnitude of *MYC* induction was approximately 3.3× greater (~117×) using intraperitoneal 4-OHT in TG mice in the current study than was demonstrated in a previous investigation utilizing a topical administration approach (~35×) [13]. These results suggest that the extent of sebaceous differentiation is MYC dose-dependent; topical 4-OHT induction, and lesser transgene activation, promotes differentiative processes, while systemic administration of 4-OHT and greater transgene activation suppresses sebaceous differentiation in the promotion of proliferation [16]. This response is also supported by both ORO staining, demonstrating smaller and more weakly staining cytoplasmic lipid droplets in TG mice relative to wt littermates, and transcript expression data, revealing attenuated *PLIN2* expression in *MYC*-overexpressing TG mice. FASN inhibition typically leads to reduced expression of lipid-associated proteins such as adipophilin (PLIN2) and the storage of lipid droplets by limiting the availability of de novo synthesized FAs which are required for droplet formation [66,67,68]. C75, specifically, has demonstrated potent effects on lipid differentiation in various stages of adipocyte maturation [69]. Increased MYC expression has been correlated with elevated FASN expression across multiple tumor types, typically demonstrating induction in the 2–10-fold range at the mRNA level, which is similar to the relative increase demonstrated in our *MYC*-overexpressing murine MGs [70,71]. These data indicate that while systemic *MYC* induction yielded target transcript expression exceeding what would likely be anticipated in spontaneous high-MYC-expressing tumoral tissue, the inductive effective of lipogenic target genes is congruent with relative fold changes in other tumor models.

There are several limitations to the current study which are characteristic of exploratory studies; ongoing studies aim to prioritize the evaluation of direct mechanistic links between FASN inhibition and lipidomic alterations (e.g., gain/loss-of-function experiments, pathway perturbations). Additionally, the use of C75 as the sole FASN inhibitor in the assessment of in vitro phenotypic responses may be confounded by the C75-mediated accumulation of upstream lipid metabolites (i.e., malonyl-CoA) which enhance β-oxidation, suggesting perturbed energy balance may contribute to cell death independently of direct on-target effects. However, to remediate some of the well-documented systemic toxicities produced by C75 in vivo, topical administration was pursued to observe only local (e.g., Meibomian gland) tissue responses. Furthermore, no FASN inhibitor, including newer generation compounds, is completely free of off-target or context-dependent effects. We also acknowledge the limited sample size utilized for lipidomic analyses, and future studies intend to expand these to permit statistical analyses in addition to pursuing more targeted LC-MS/MS approaches to quantify individual lipid species. Finally, while transgene induction was demonstrably much more efficient in response to systemic (IP) 4-OHT administration than topical, we acknowledge that the approximately 35x increase in *MYC* expression achieved following topical administration is likely a more accurate model of the cancer biology presented by spontaneous high-MYC-expressing tumors. However, mild to moderate blepharitis was observed in several mice in response to topical 4-OHT induction followed by a course of topical C75 treatment, and systemic administration of 4-OHT was elected to avoid unnecessary pain and discomfort.

## 5. Conclusions

Metabolic insights into SebCA tumor biology will pave the way for innovative and effective targeted therapies. Although lipid metabolism is often dysregulated in cancers, the mechanisms of MYC-mediated lipogenic alterations in SebCA remain unresolved. Employing phenotypic assays and untargeted mass spectrometry, we characterized the effects of FASN inhibition on the lipid metabolism of MYC-driven SebCA cell lines. These findings provide compelling evidence that MYC is a major regulator of lipogenesis and FA saturation, which are processes that are critical for both the initiation and maintenance of cancer. Consequently, targeting lipid synthesis may hold potential as an effective therapeutic approach against high-MYC-expressing SebCA.

## Data Availability

The original contributions presented in the study are included in the article. Further inquiries can be directed to the corresponding author.

## Supplementary Materials

The following supporting information can be downloaded at https://www.mdpi.com/article/10.3390/cancers18020349/s1, R script for determining relative abundance of saturated and unsaturated fatty acids; Figure S1: Apoptosis Responses to FASN Inhibition In Vitro; Figure S2: Relative abundance of individual lipid metabolites isolated from MYC and FASN-inhibited HMGECs by lipid class; Figure S3: Relative abundance of individual lipid metabolites isolated from MYC and FASN-inhibited SebCA01 cells by lipid class; Figure S4: Relative abundance of individual lipid metabolites isolated from MYC and FASN-inhibited SebCA02 cells by lipid class; Figure S5: Relative abundance of individual lipid metabolites isolated from MYC and FASN-inhibited SebCA03 cells by lipid class; Figure S6: Differentiative and Proliferative Sequelae of FASN Inhibition In Vivo.

## Abbreviations

The following abbreviations are used in this manuscript:

SebCA	Sebaceous carcinoma; malignant neoplasm arising from sebaceous glands.
MG	Meibomian gland; specialized sebaceous glands of the eyelid responsible for synthesis and secretion of the lipid portion of the tear film.
HMGECs	Human Meibomian gland epithelial cells; immortalized, non-neoplastic meibocyte line
MYC	c-MYC; protooncogene.
FASN	Fatty acid synthase; enzyme which catalyzes de novo fatty acid synthesis.
SREBP-1	Sterol regulatory binding protein-1; transcription factor regulating lipid biosynthesis.
PPARγ	Peroxisome proliferator-activated receptor gamma; transcription factor involved in lipogenesis.
FA	Fatty acid; carboxylic acid consisting of a long hydrocarbon chain, attached to a carboxyl group.
SFA	Saturated fatty acid; fatty acid containing no carbon–carbon double bonds (C=C).
UFA	Unsaturated fatty acid; fatty acid containing at least one C=C.
TG	Transgenic; K14MycER mouse.
wt	Wildtype; C57B6 mouse.
4-OHT	4-hydroxytamoxifen; selective estrogen receptor modulator and TG inducer.
