# Supplementary material for: Fatty Acid Synthase as a Potential Metabolic Vulnerability in Ocular Adnexal Sebaceous Carcinoma
Source: Cancers (Basel). 2026 Jan 22;18(2):349. doi: 10.3390/cancers18020349 (PMC12839348; doi:10.3390/cancers18020349)
Supplement: Supplementary file 1 [file cancers-18-00349-s001.zip › cancers-4078406-supplementary.pdf]

R script for determining relative abundance of saturated and unsaturated fatty acids:

```
---
title: "Saturation_Analysis"
---

{r setup, include=FALSE}
knitr::opts_chunk$set(echo = TRUE)

{r}
# Load the data set
library(readxl)
# yourFile <- read_excel(file path)
View(yourFile)

data_sorted <- yourFile %>%
  # Extract lipid class
  mutate(Lipid_Class = str_extract(Lipid, '^[A-Za-z]+'),

         # Extract chain info about carbon bonds ie '18:1'
         Chain = str_extract(Lipid, '[0-9]+:[0-9]+'),

         # extract carbon ct and double bonds
         Carbons = as.numeric(str_extract(Chain, '^[0-9]+')),
         Double_Bonds = as.numeric(str_extract(Chain, '(?<=:)[0-9]+'))) %>%

# Arrange by CLASS then SATURATION (double bonds)
arrange(Lipid_Class, Double_Bonds)

# Save output as csv
write_csv(data_sorted, 'sorted_by_saturation.csv')
head(data_sorted)

data4 <- read_csv("sorted_by_saturation.csv", col_types = cols(
  Formula = col_character(),
  Lipid = col_character(),
  DMSO = col_double(),
  MYCi = col_double(),
  MI6 = col_double(),
  10074 = col_double(),
  C75 = col_double(),
  Lipid_Class = col_character(),
  Chain = col_character(),
  Carbons = col_double(),
  Double_Bonds = col_double(),

)) %>%
  mutate(Lipid_Class = str_extract(Lipid, "^[A-Za-z]+"),
         Chain = str_extract(Lipid, "[0-9]+:[0-9]+"),
```

```
      Carbons = as.numeric(str_extract(Chain, "^[0-9]+")),
      Double_Bonds = as.numeric(str_extract(Chain, "(?<=:)[0-9]+")))

data_long <- data4 %>%
  pivot_longer(cols = c(DMSO, MYCi, MI6, 10074, C75),
               names_to = "Treatment",
               values_to = "Abundance")

#step 3
data_long <- data_long %>%
  mutate(Saturation = if_else(Double_Bonds == 0, "Saturated", "Unsaturated"))
write.csv(data_long, file = "data_long.csv", row.names = FALSE)

#step 4
saturation_summary <- data_long %>%
  group_by(Treatment, Saturation) %>%
  summarise(
    Total_Abundance = sum(Abundance, na.rm = TRUE),
    Mean_Abundance = mean(Abundance, na.rm = TRUE),
    .groups = "drop"
  )
write.csv(saturation_summary, file = "saturation_summary.csv", row.names = FALSE)

# plot abundance
ggplot(saturation_summary, aes(x = Treatment, y = Total_Abundance, fill = Saturation)) +
  geom_col(position = "dodge") +
  theme_minimal() +
  ylab("Total Abundance") +
  ggtitle("Saturated vs Unsaturated Lipids Across Treatments")
```

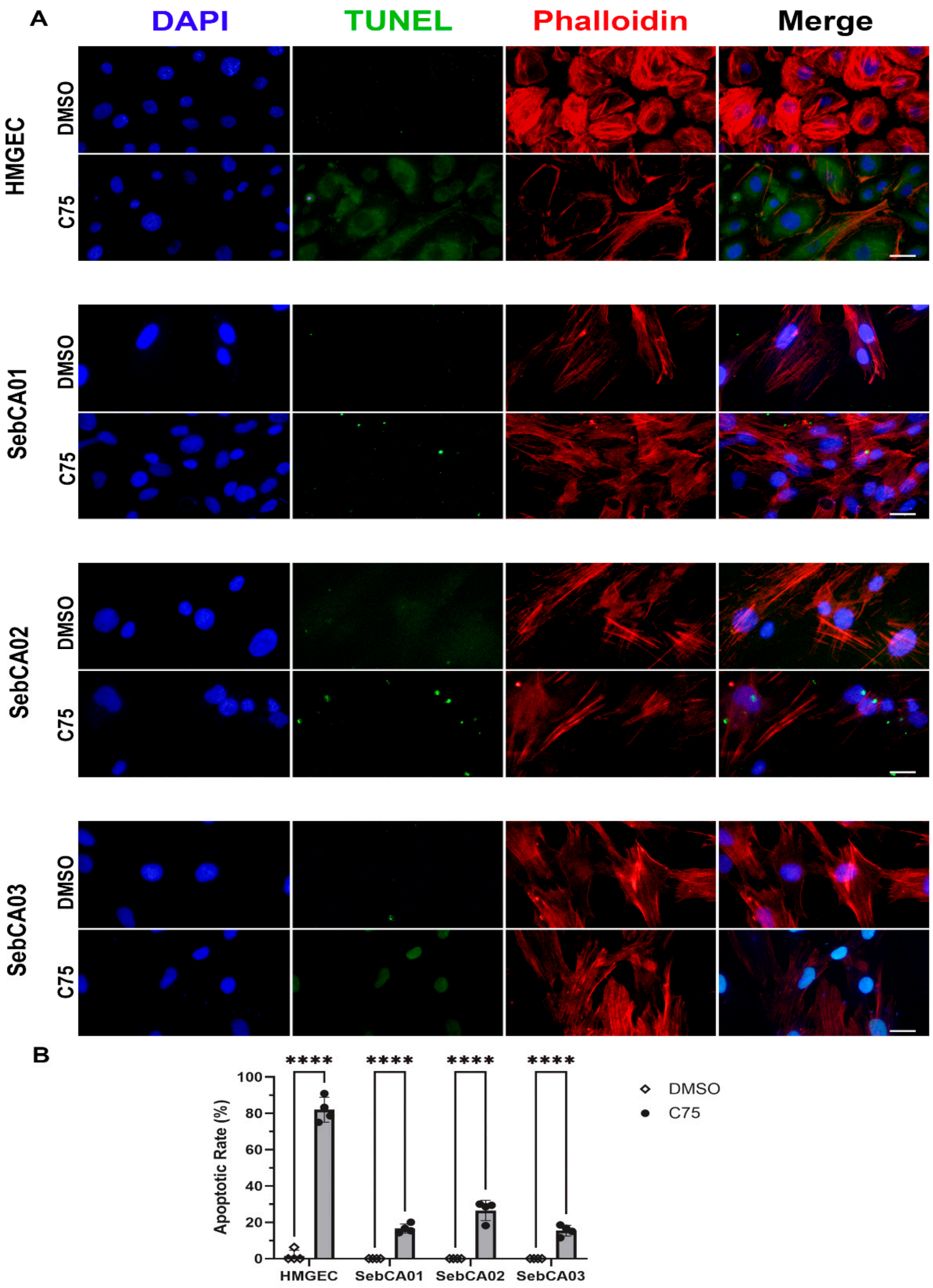

Supplementary Figure S1: Apoptosis Responses to FASN Inhibition *In Vitro*. (**A**)The number of TUNEL-positive nuclei (yellow green, merged), indicating DNA fragmentation and apoptosis, were increased across all cell lines in response to FASN inhibition relative to vehicle controls. HMGECs exhibited the greatest apoptotic cell death. TUNEL: Alexa Fluor 488. Phalloidin: Alexa Fluor 555. DAPI: blue. Scale bar: 10 μm for all panels. (**B**) Quantification of TUNEL-positive cells demonstrated a significant induction in mean apoptotic rate in C75-treated cells relative to DMSO in all cell lines. **** $p \leq 0.001$.

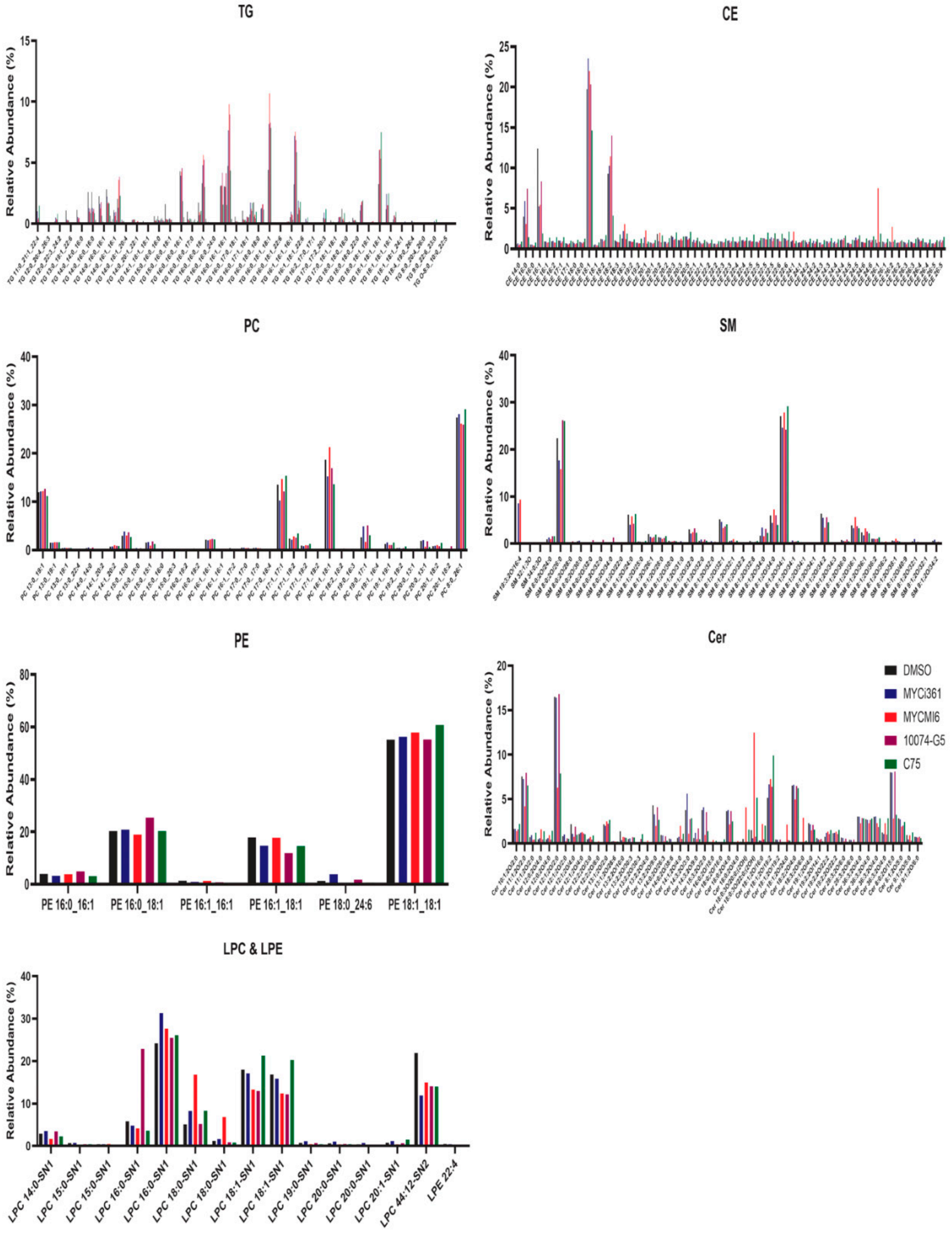

Supplementary Figure S2: Relative abundance of individual lipid metabolites isolated from MYC (MYCi361, MYCMI6, and 10074-G5) and FASN-inhibited (C75) HMGECs by lipid class. TG: triacylglycerol; PC: phosphatidylcholine; PE: phosphatidylethanolamine; LPC & LPE: lysophosphatidylcholine and lysophosphatidylethanolamine; CE: cholesterol ester; SM: sphingomyelin; Cer: ceramide.

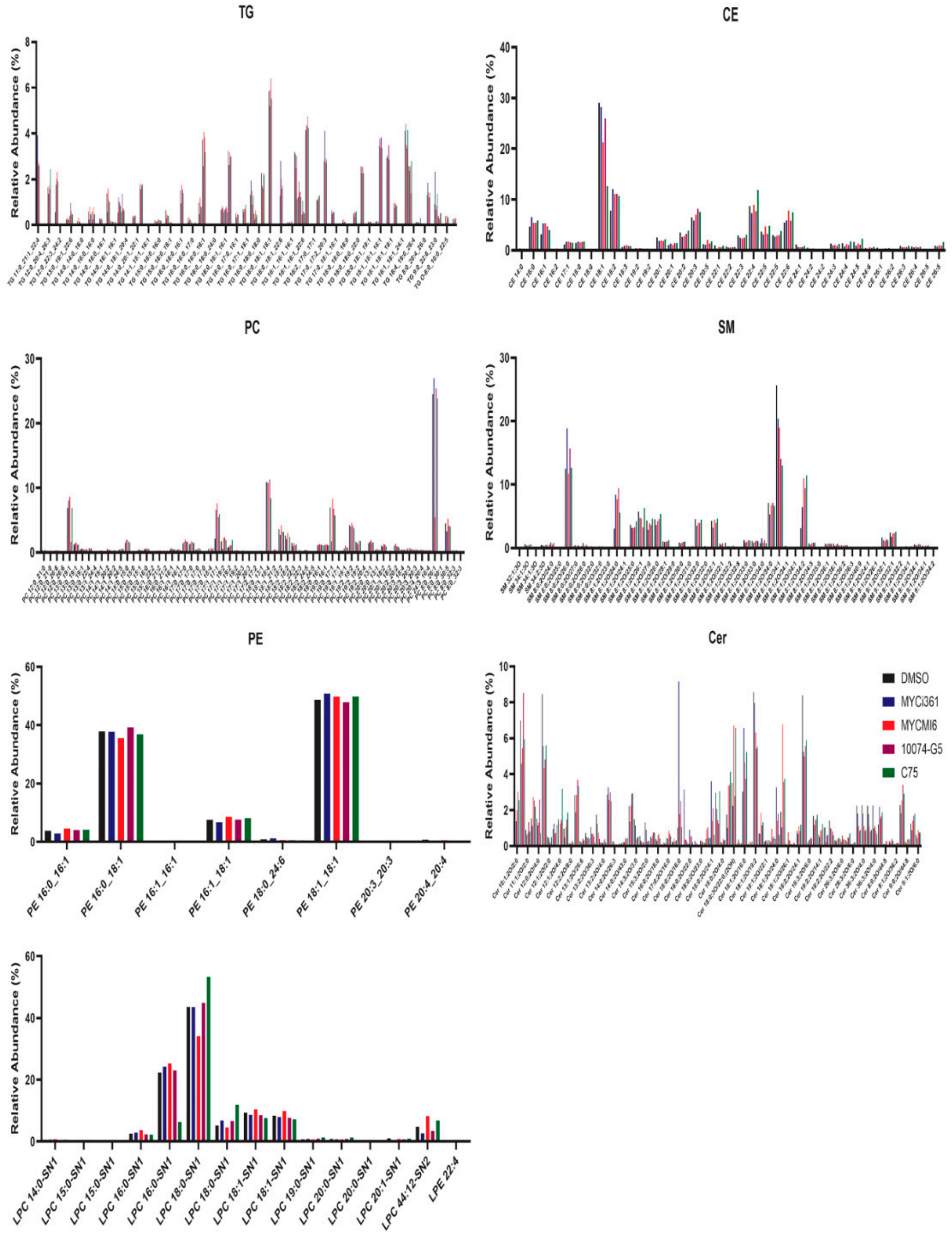

Supplementary Figure S3: Relative abundance of individual lipid metabolites isolated from MYC (MYCi361, MYCMI6, and 10074-G5) and FASN-inhibited (C75) SebCA01 by lipid class. TG: triacylglycerol; PC: phosphatidylcholine; PE: phosphatidylethanolamine; LPC & LPE: lysophosphatidylcholine and lysophosphatidylethanolamine; CE: cholesterol ester; SM: sphingomyelin; Cer: ceramide.

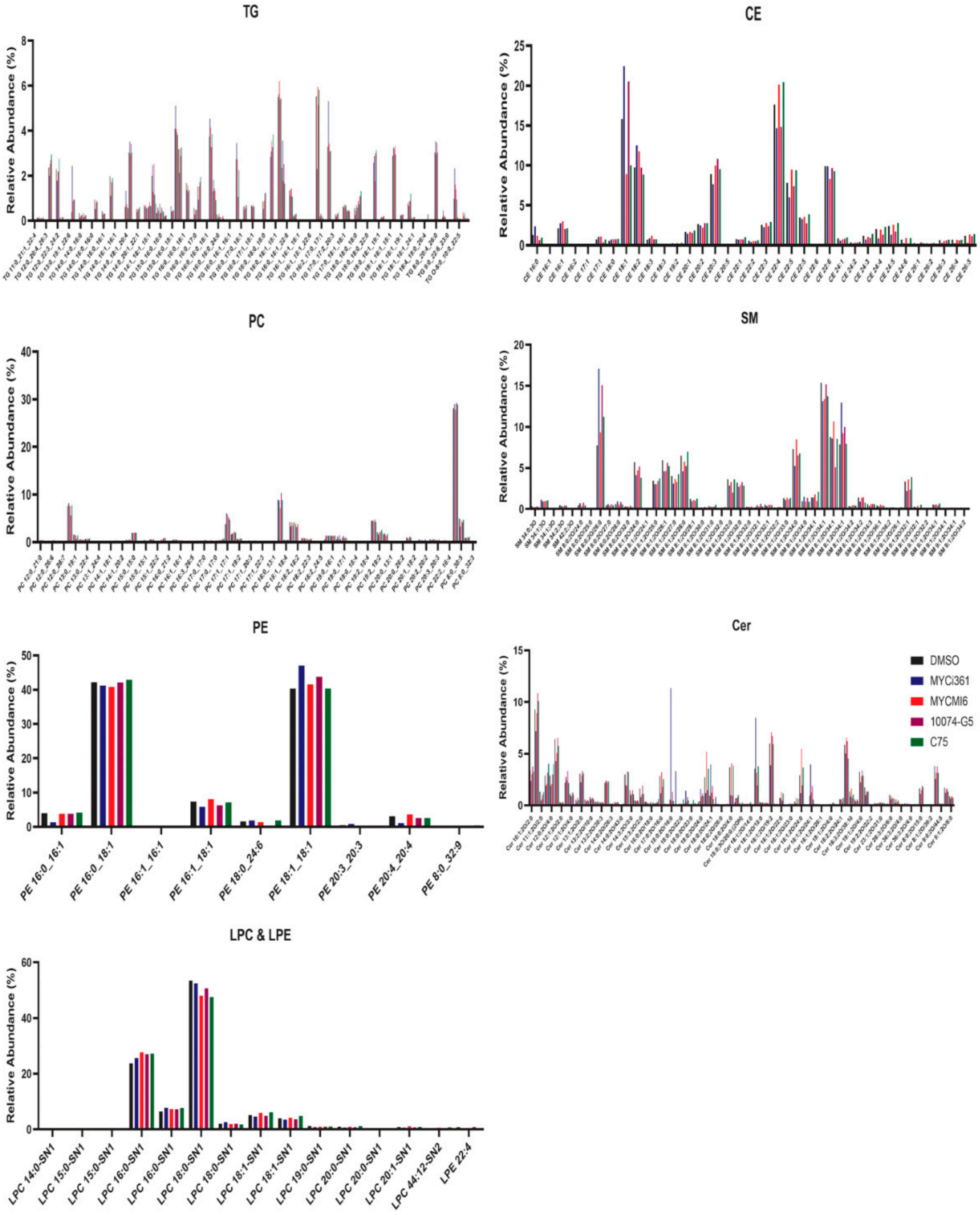

Supplementary Figure S4: Relative abundance of individual lipid metabolites isolated from MYC (MYCi361, MYCMI6, and 10074-G5) and FASN-inhibited (C75) SebCA02 by lipid class. TG: triacylglycerol; PC: phosphatidylcholine; PE: phosphatidylethanolamine; LPC & LPE: lysophosphatidylcholine and lysophosphatidylethanolamine; CE: cholesterol ester; SM: sphingomyelin; Cer: ceramide.

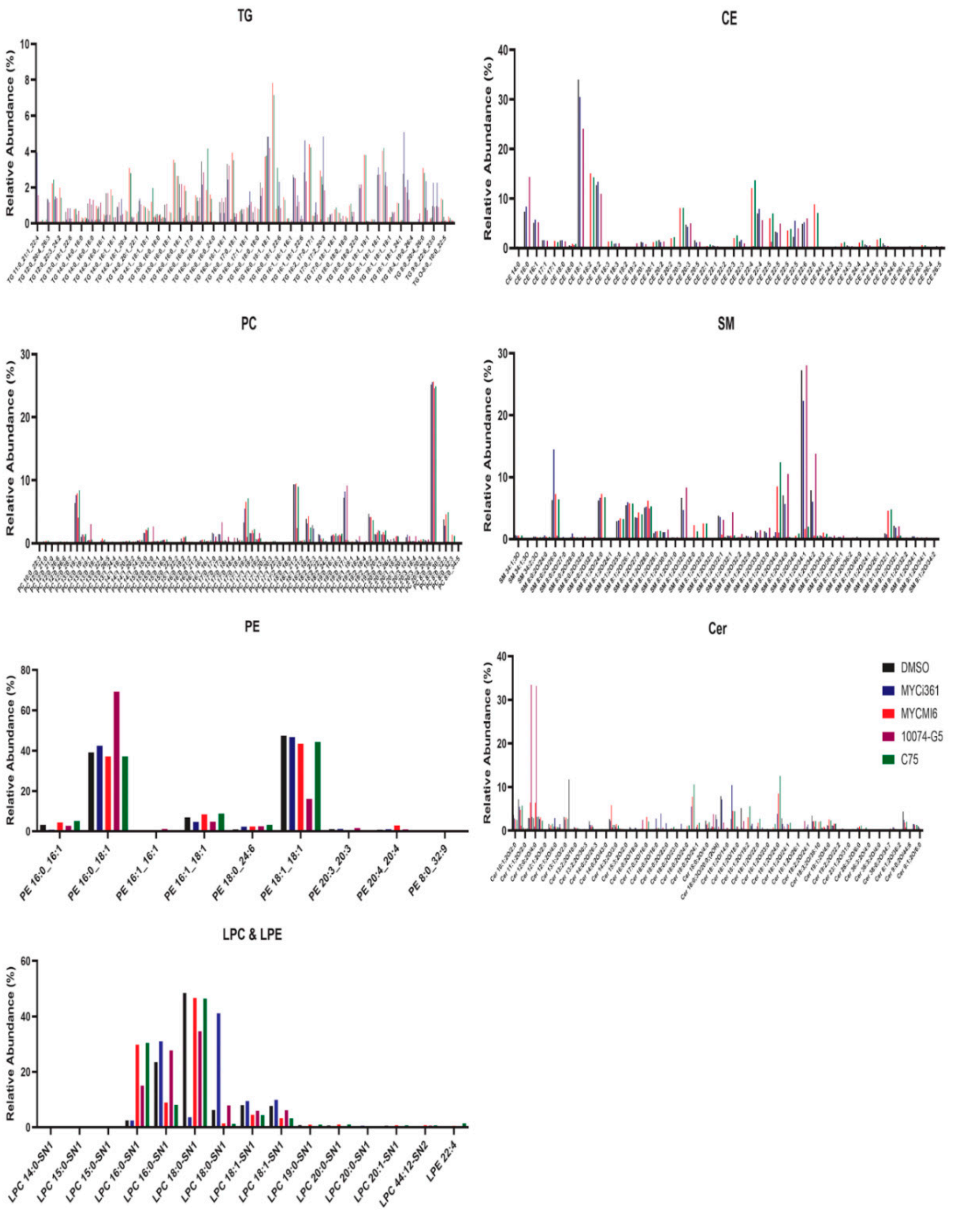

Supplementary Figure S5: Relative abundance of individual lipid metabolites isolated from MYC (MYCi361, MYCMI6, and 10074-G5) and FASN-inhibited (C75) SebCA03 by lipid class. TG: triacylglycerol; PC: phosphatidylcholine; PE: phosphatidylethanolamine; LPC & LPE: lysophosphatidylcholine and lysophosphatidylethanolamine; CE: cholesterol ester; SM: sphingomyelin; Cer: ceramide.

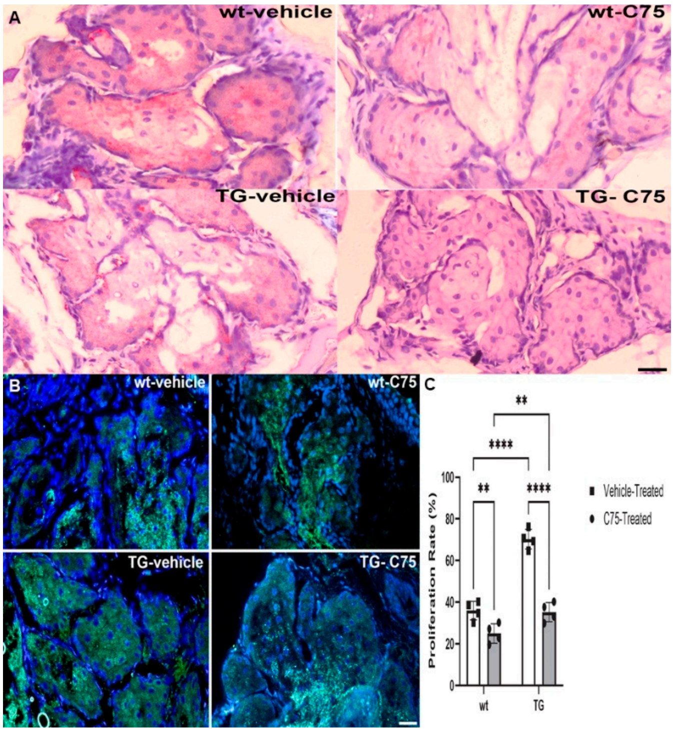

Supplementary Figure S6: Differentiative and Proliferative Sequelae of FASN Inhibition *In Vivo*. Representative histochemical and immunofluorescent-stained images frozen sections of murine tissues. (**A**) Conditionally *MYC*-overexpressing transgenic (TG) Meibomian glands exhibited diminished sebaceous differentiation when stained with Oil-Red-O (ORO: red orange) relative to wildtype (wt) littermates. Sebaceous differentiation was further attenuated in response to C75 treatment. Scale bar: 50 µm**.** (**B**) 4-OHT-induced conditionally *MYC*-overexpressing TG glands exhibited upregulated proliferative responses (BrdU: Alexa Fluor 488, green) relative to wt littermates and when compared to C75-treated eyelids. DAPI: blue. Scale bar: 50 µm (**c**) Quantification of BrdU-positive cells demonstrated a significant reduction in the mean proliferation rate in C75-treated eyelids relative to contralateral controls. ** $p < 0.01$, **** $p \leq 0.0001$.
